# Addressing the Inertia: A Holistic Approach to Diabetic Foot Evaluation

**DOI:** 10.7759/cureus.37186

**Published:** 2023-04-05

**Authors:** Jayshree Swain, Abhay Kumar Sahoo, Pooja A Jadhao, S.L. Sravya, Brij R Teli

**Affiliations:** 1 Endocrinology, Diabetes and Metabolism, Institute of Medical Sciences and SUM Hospital, Bhubaneswar, IND

**Keywords:** diabetic foot risk evaluation, foot care, diabetic foot examination, diabetic neuropathy evaluation, diabetic foot

## Abstract

Diabetic foot is a well-known complication with considerable morbidity and mortality related to the diabetic population. Neuropathy, deformity, infection and ischemia are important contributors to the pathogenesis of diabetic foot ulcers. A multidisciplinary team approach by physicians, nursing staff, diabetic educators and the caregiver as well as close monitoring of feet by the patient himself can prevent foot-related complications. Proper foot care, foot hygiene, annual foot examination and the correct choice of footwear are the main elements in preventing foot problems like deformity, ulceration and amputations. Physicians play a key role in the early detection and prevention of foot problems. Foot evaluation is the most neglected part of physical examination in diabetic patients. This inertia is evident in both physicians and patients. Emphasis on visual inspection and physical foot examination at every visit may address the morbidity and mortality due to diabetic neuropathy and vasculopathy. This article will highlight extensively history taking and foot examination in the diabetic foot clinic. Optimal glycemic control, simple foot care practices, knowledge and appropriate footwear use play an important role to reduce the disease burden.

## Introduction and background

Among the chronic complications of diabetes, diabetic foot (DF) problems are more frequent, with an impact on the economic burden in the diabetic population [1]. The major reasons for the development of DF include longer duration of uncontrolled diabetes, diabetic peripheral neuropathy and peripheral vascular disease [2]. The diabetic population should have regular follow up with their physician. A detailed history and good general and systemic examination including foot examination are an integral part of a fruitful visit. Foot examination is an important aspect of diabetes management, which should never be missed.

Most people with diabetes do not have any primary foot-related complaints. Rather foot problems are usually silent and asymptomatic in 50% of cases [3]. Many times, a wound or an ulcer in the patient’s foot is noted by relatives or detected during a routine check-up. Longstanding uncontrolled diabetes, distal symmetrical peripheral neuropathy, loss of protective sensation in the feet and repeated trauma lead to deformities and painless wounds [4,5]. Infection and ischemia have an additional role in the causation of DF ulcers [6]. A delay in attention to the foot ulcer may lead to major surgery or amputation.

Above all, the economic burden of DF is too high, owing to loss of productivity or absenteeism from work, hospitalization and the cost of treatment [7]. It is reported that good diabetes control and proper foot care have resulted in a 50% decrease in foot amputations in the last 20 years [8]. Education of patient, nurse, caregiver and physician along with close monitoring prevent foot complications by 49 to 85% [9]. Proper foot care and hygiene, appropriate footwear, visual inspection of feet at every clinic visit and comprehensive annual feet exam by health care professionals are key to healthy diabetic feet [10].

During a foot examination, the following factors must be considered: a detailed history, clinical examination, neuropathy evaluation, peripheral artery disease assessment, and foot care advice. They will all be addressed in this review.

## Review

History

A detailed history should be elicited from all patients. Duration and control of diabetes should be enquired about. History of the accident, injury, fracture, sprain, infection, surgery, previous ulcerations, amputation, revascularization procedures or nail avulsion (traumatic or surgical) or barefoot walking practice needs to be enquired about [11]. Enquiry about positive neuropathic symptoms like burning, tingling, pin pricking and electric shock-like sensation and negative symptoms like numbness to be done along with the history of diurnal variation as neuropathic symptoms tend to be worse at night [12]. History of a cotton wool-like sensation on the soles while walking or slipping of footwear while walking needs to be elicited. Any history of gait imbalance also needs to be enquired about.

History should also include that of occupation, diet, smoking, alcohol intake, drug history specifically those causing neuropathy (like antiepileptic drugs, antitubercular drugs, antiarrhythmic drugs, etc.), any associated illness, comorbidities, claudication pain, rest pain and general care of the foot.

Autonomic nervous system involvement is also commonly seen in diabetes, so the history of dryness of feet with the presence of heel cracks should be enquired about as it denotes the presence of autonomic dysfunction. In addition, history regarding the autonomic involvement of other systems like cardiovascular, gastrointestinal and genitourinary dysfunction can be elicited. These symptoms include syncope, hypotension, resting tachycardia, the fullness of the abdomen, altered bowel pattern, faecal incontinence, straining and incomplete voiding of urine, gustatory and differential sweating and extreme diaphoresis.

Clinical examination

A thorough foot examination, after removing shoes and socks, should be done in a well-lit room at each visit to diagnose any complications. Some patients may not have any neuropathic symptoms or complaints, so the next important step is the examination of the foot and lower limbs and eliciting the signs of neuropathy, evidence of peripheral arterial disease and any abnormalities or deformities. The examination begins as soon as the patient walks inside the room and gait can be evaluated by the patient’s walk. Asking the patient to remove shoes and socks ensures a thorough foot examination.

Skin

The colour of the skin of the feet and legs should be checked. Any change in colour like redness, blackish discolouration or pigmentation of skin should be noted. Dryness of feet, legs, cracks, fissures and scaly or thickened skin which are the signs of neuropathy and poor circulation should be noted. Cracks and fissures may act as a portal of entry of infection [13]. The temperature should be checked with the dorsum of the hands or sophisticated devices like dermal thermometers. A temperature difference of at least two degrees more than the contralateral foot should be evaluated further for any underlying cellulitis, infection or vascular compromise. The hard skin areas or high-pressure points, callus, corns, old healed injury marks and surgical scars should also be checked. If due care is not given to callus on the plantar aspect, malleolus or on bony prominences, it may lead to infection, haemorrhage or ulceration [14]. Heloma durum or hard corns generally appear on the tips of toes or plantar aspect due to inappropriate footwear. Heloma molle or soft corns appear in the interdigital space due to pressure of the adjacent toe phalanges. They tend to retain moisture and are predisposed to fungal infections. Corns may be multiple or single, recurrent, deep or superficial, painless or painful. Painful and infected corn needs intervention. Any bad odour from the feet should be taken note of as poor foot hygiene and web space fungal infections give rise to it [15]. The skin of the legs and feet should be checked for blisters which may appear spontaneously or after exposure to heat or ill-fitting footwear. They are often unnoticed if a person has peripheral neuropathy and may lead to complications like infection and ulcers. Loss of dermal hair on the legs may be an indication of peripheral neuropathy and poor perfusion. Guttering between the metatarsals due to muscle wasting should be checked.

Web Spaces

Interdigital fungal infections are common in individuals with diabetes. Major contributory factors are clawing and crowding of toes (which is mainly due to motor neuropathy) and inappropriate foot hygiene practices (due to associated factors like impaired vision, obesity, advancing age and lack of awareness regarding the same) [16]. Inspection of web spaces is mandatory to rule out infections as well as to ensure that the patient is compliant with foot hygiene.

Shape/Deformity

**Figure 1:**
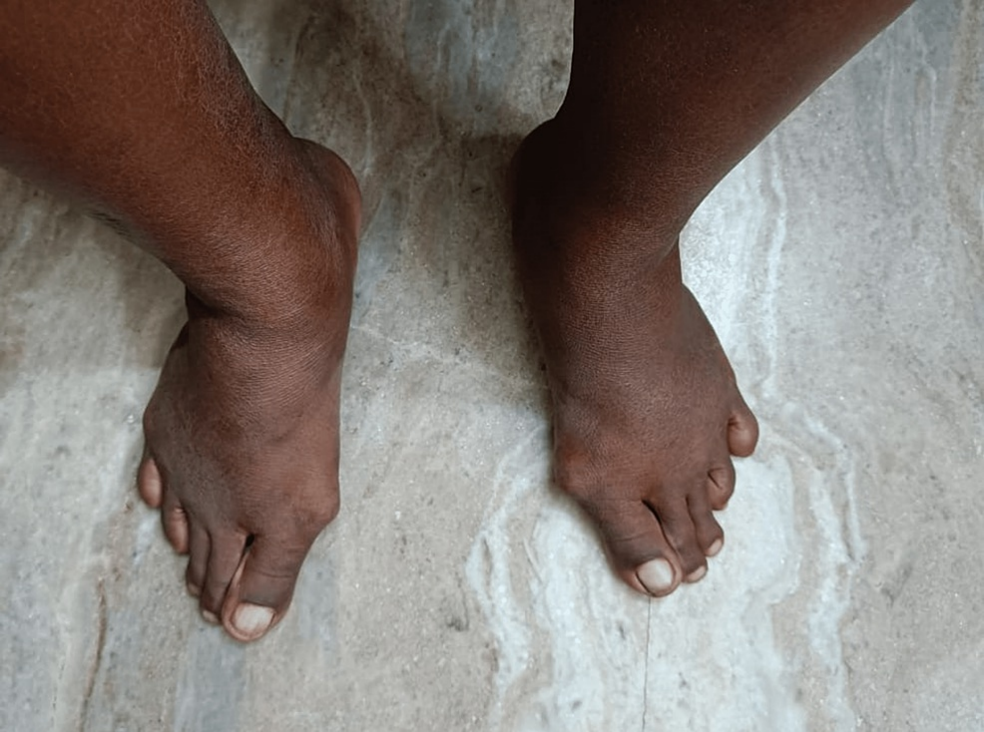
Hallux valgus Photograph was taken with patient's consent.

**Figure 2:**
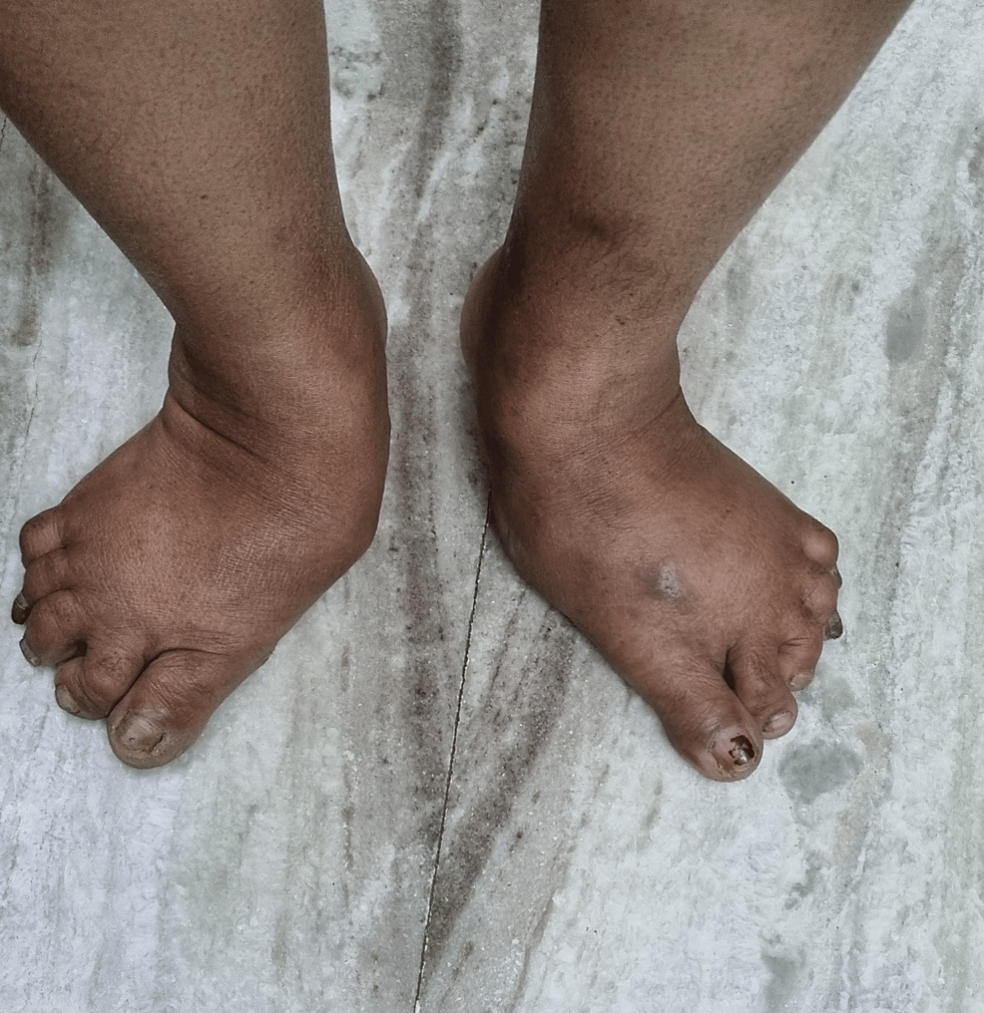
Charcot's osteoarthropathy Photograph was taken with patient's permission.

Motor and autonomic neuropathy cause deformities, and structural changes in the shape of the foot leading to abnormal pressure points and bony prominences, which further cause skin ulceration gradually [17]. Sensory loss due to peripheral neuropathy exacerbates the development of ulceration. An imbalance between the flexors and extensors in the foot leads to clawing of toes, hammer toes, mallet toes, hallux valgus (Figure 1) and crowding or overlapping of toes. Clawing of toes is dorsiflexion of the tarsometatarsal joints with plantarflexion of distal and proximal interphalangeal joints, hammer toes are plantarflexion of proximal interphalangeal joints whereas mallet toes are plantarflexion of distal interphalangeal joints. Any change in shape, loss of the arch of the foot, low or high arch foot or bony prominences like bunion need to be checked. The prevalence of advanced foot deformities like Charcot foot (Figure 2) varies and can be as much as 9.8% in diabetic patients with neuropathy and is usually painless [18]. Charcot foot is a degenerative arthropathy precipitated in an insensate foot by repeated microtrauma. Identification of chronic Charcot foot is essential to prevent ulceration and infection in the predisposed foot. The presence of foot drop should probe the palpation of peripheral nerves to rule out any tenderness or thickening.

Nails

Brittle, ingrown nails, dystrophic nails or onychomycosis are commonly found in individuals with diabetes. Surgical intervention is warranted for ingrown nails as they may lead to paronychia or abscess. The presence of onychomycosis, onychocryptosis, onychodystrophy or onychogryphosis should be evaluated. Nail trimming technique should be checked and if wrong, the correct way to trim the nails should be explained.

Peripheral Pulses

The patient experiences claudication-like pain (pain in the legs while walking which is relieved with rest) if blood flow is hampered. Mainly two arteries are to be palpated, the dorsalis pedis artery and the posterior tibial artery. The dorsalis pedis artery is palpated on the base of the navicular bone just lateral to the extensor hallucis tendon and the posterior tibial artery is palpated just behind the medial malleolus, midway between the medial malleolus and Achilles tendon. Pulse may be weak, normal, bounding, absent or may not be felt due to swelling. Weak or absent pulse and cold feet indicate peripheral vascular disease. Any abnormality should be evaluated further by Ankle Brachial Index (ABI) or by a vascular Doppler study. In addition, palpation of the popliteal artery can be undertaken behind the knee joint with the patient’s knee slightly flexed. Femoral artery palpation is to be performed when required.

Neuropathy evaluation

Evaluation of pain, touch and temperature along with vibration and joint position should be carried out. Pain sensation is checked with the help of a pinprick. Testing should begin from the distal tips of the toes and progress proximally, the exact site of demarcation where the sensations are normal as compared to earlier diminished sensations should be noted. Similarly, touch sensation should be evaluated with the help of a cotton wisp. The tip of the wisp of cotton should be touched with a light stroke at precise sight without rubbing the surrounding skin. A few false or sham strokes should also be included to assess the patient. Temperature sensation can be tested with the help of test tubes filled with hot and cold water or the handle of the tuning fork. Hot and cold test tubes are to be applied to the skin of the feet alternately and the patient was asked whether he perceived a hot or cold sensation.

The 128 Hz tuning fork can be used to evaluate the vibratory perception [19]. A tuning fork should be set in vibration and kept on the metatarsal phalangeal joint of the big toe. The subject is asked for a response as to whether he can feel the vibration and also when it stops. After that, it is immediately placed on the wrist of the subject. If the vibration is still felt on the wrist, it indicates the patient has impaired vibratory perception.

**Figure 3:**
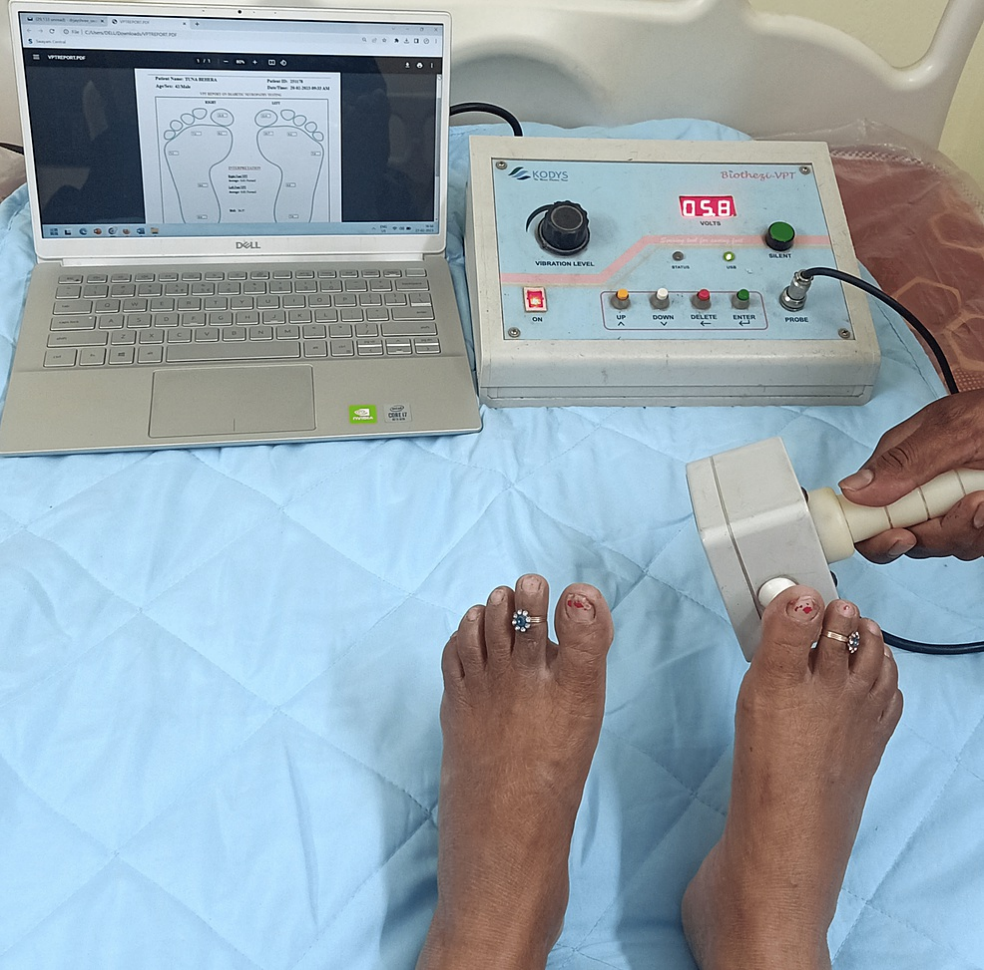
Biosthesiometer Photograph was taken with patient's permission.

A Biosthesiometer measured vibration perception threshold (VPT) can also denote the risk of ulceration in the patient. A hand-held probe is applied to fixed six sites on the plantar foot surface and the vibration threshold is progressively increased (Figure 3). The volts at which the patient perceives the vibration are noted down. A VPT of 15-25 volts and >25 volts denotes moderately and severely impaired vibration perception respectively [20].

Testing for proprioception or joint position is carried out by rapid dorsiflexion and plantarflexion of the proximal interphalangeal joint of the great toe followed by questioning the patient about the position of the toe. If the patient correctly answers ‘down’ when the toe is plantarflexed the joint position sensation is intact.

Testing for muscle strength and the ankle reflex also forms an integral part of foot examination in diabetic individuals. The quadriceps femoris and the tibialis anterior are the main muscles to be evaluated. Dorsiflexion of the foot at the ankle joint assesses the tibialis anterior muscle. Grading for muscle strength is done with grade 5 being normal strength against full resistance, grade 4 being a movement against gravity with some resistance and grade 3 being a movement against gravity but without resistance, grade 2 power is the presence of flickering movement and grade 1 power denotes no movement of limbs. Quadriceps femoris is assessed with the patient in a seated position and asked for an extension of the leg.

Ankle reflex testing is done with the help of a reflex hammer. The patient is positioned supine with the knee joint flexed and the hip externally rotated. The patient's foot is slightly dorsiflexed by the examiner and the tendon of the gastrocnemius muscle is struck by the flat part of the hammer. The contraction of the muscle denotes the presence of an ankle jerk. If ankle reflex is absent, it is to be rechecked with reinforcement manoeuvres.

**Figure 4:**
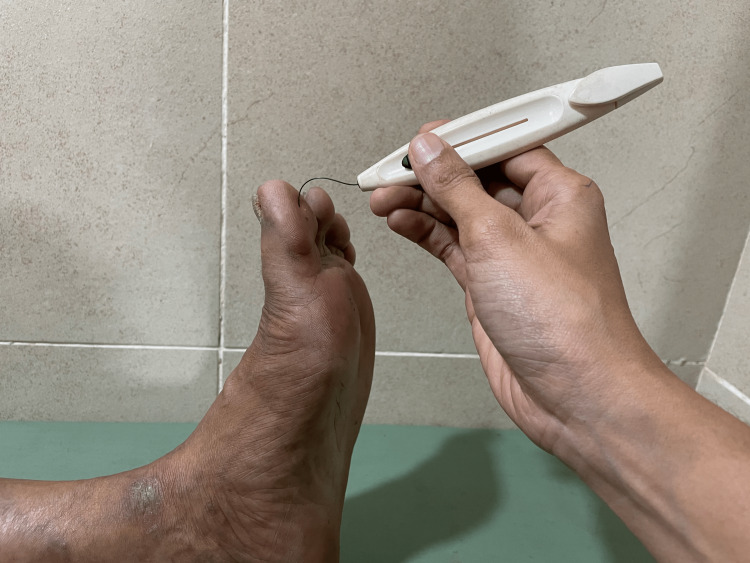
10 g monofilament test Photograph was taken with patient's permission.

Other simple bedside instruments that can be used for neuropathy testing include 5.07 Semmes-Weinstein Monofilament. A 10 g Semmes-Weinstein Monofilament is used to check protective sensation in the feet. A 10 g monofilament is kept perpendicular to the skin on the big toe, metatarsal heads, instep and heel, and pressure is applied till it buckles to form a C shape which exerts 10 g weight (Figure 4). If the person is able to feel the touch, then his protective sensation is intact. The inability to perceive sensation at two sites denotes loss of protective sensation (LOPS). Insensitivity to the 10g monofilament implies increased foot ulceration risk by 10-fold [21].

Other tests to evaluate feet for high-pressure points include using techniques like podoscope scanning, carbon paper imprint of the foot, foam marks and in-shoe measurement systems. Podoscan assesses plantar pressure distribution. It evaluates the dynamic pressures of the foot and areas of increased plantar pressure that can lead to deformity. Early detection of distorted distribution of plantar pressures can identify and prevent foot deformities. It can also aid in designing custom footwear based on plantar pressure distribution. Newer modalities like electronic foot pressure mapping systems are also utilized for the same.

Clinical scoring systems for neuropathy detection

Different clinical scoring systems can be used for the purpose of screening for diabetic peripheral neuropathy. The screening is based on symptoms described by the patient or on the clinical examination findings elicited by the examiner. These scoring systems are a composite of various signs and symptoms and thus have a better diagnostic value than individual neurological examination findings [22]. A few examples of scoring systems specific for screening diabetic peripheral neuropathy are Diabetic Neuropathy Examination (DNE), Diabetic Neuropathy Symptom Score (DNS), Michigan Neuropathy Screening Instrument (MNSI), Neuropathy Disability Score (NDS), Neuropathy Impairment Score (NIS), Neuropathy Impairment Score in Lower Limbs (NIS-LL), Toronto Clinical Scoring System (TCSS). DNS and revised NDS are the most commonly used tools.

The DNS score is a simple tool comprising four items that include unsteadiness of gait, burning or aching sensation, pricking sensation and numbness. The presence of a symptom adds one point to the scoring system and a score of one or more is indicative of the presence of neuropathy [23].

The NDS comprises 35 items for each side and is cumbersome for outpatient evaluation. In addition, some of the items in the scoring system were found to not be related to neuropathy. Hence a revised NDS with a total of 10 points was adopted. It consists of an evaluation of pin-prick sensation, vibration sensation with a 128 Hz tuning fork and temperature sensation with the cold handle of the tuning fork on both the great toes. The presence of these sensations adds zero points while their absence or reduced sensation adds one point. Ankle reflex testing on both sides is done, its presence adds zero points, presence with reinforcement adds 1 point and absence adds 2 points. A score of six or more favours the presence of diabetic peripheral neuropathy [24]. Combining the symptom score and signs score had a sensitivity of 71.1% and a specificity of 90% in a validation study of the combined scores. It had positive and negative predictive values of 57.14% and 94.32% [25].

DNE is adapted from NDS and comprises only eight items of the original 35. Here testing is done only on the right side of the body. Muscle strength, reflexes and superficial sensations are evaluated. Out of a maximum of 16 points, a score of 3 or more is considered to be positive for neuropathy [26].

MNSI is another popular tool used in diabetic peripheral neuropathy screening. It comprises a self-administered questionnaire and clinical examination. A score of 7 out of 15 questions in the questionnaire part is defined as abnormal and a score of 2.5 or more in the foot examination part is positive for neuropathy [27].

TCSS is another validated screening tool for diabetic neuropathy which includes three parts - symptom score, reflexes score and sensory tests score. A score of 6 to 8 points classifies DPN as mild, 9 to 11 as moderate and 12 to 19 as severe [28]. In a validation study of TCSS, it showed a significant negative correlation with sural nerve fibre density (R2 = 0.256, P < 0.0001) [29]. Details of the individual components of some common clinical scoring systems are mentioned in Table 1.

**Table 1:** Clinical scoring symptoms for diabetic neuropathy evaluation

Scoring system	Components	Scoring	Presence of diabetic neuropathy
Diabetic Neuropathy Symptom Score (DNS)	Unsteadiness of gait, burning/aching sensation, pricking sensation, numbness	Present = 1 Absent = 0	Score of 1 or more out of 4
Revised Neuropathy Disability Score (NDS)	Pin-prick sensation, vibration sensation (128 Hz tuning fork), temperature sensation. Ankle reflex (both sides tested)	Present = 0 Absent = 1 Absent = 2 Present with reinforcement = 1 Present =0	Score of 6 or more out of 10
Diabetic Neuropathy Examination (DNE)	Extension of quadriceps muscle Dorsiflexion of tibialis anterior muscle Triceps reflex pinprick sensation (index finger). Pinprick sensation (great toe), touch sensation (great toe), vibration sensation (great toe), joint position at the great toe (only right side tested)	Normal = 0 Mild or moderate deficit = 1 Absent or Severe deficit = 2	Score of 3 or more out of 16
Michigan Neuropathy Screening Instrument (MNSI)	Self-administered questionnaire: History of foot ulcer, cracks, amputations, lower limb cramps and muscle weakness. History of positive sensory symptoms (pain, temperature, tingling), history of negative symptoms (numbness). Foot examination: Appearance of the foot (cracks), foot ulcers, ankle reflex testing and vibration testing (128 Hz tuning fork)	Yes = 1 No = 0 Yes = 1 No = 0 Absent = 1 Present with reinforcement = 0.5 Present = 0	Score of 7 out of 15 questions; Score of 2.5 or more out of 8
Toronto clinical scoring system (TCSS)	Symptoms: Pain, numbness, tingling weakness, ataxia upper limb symptoms. Reflex testing: Ankle reflex, knee reflex, sensory testing: Pinprick sensation, light touch, temperature sensation, vibration sensation, position sense	Present = 1 Absent = 0 Absent = 2 Present with reinforcement = 1 Present =0 Present = 0 Absent = 1	Score of 6 to 8 -mild DPN 9 to 11 -moderate DPN 12 to 19 - severe DPN

Tests for evaluation of peripheral arterial disease

Foot examination remains incomplete without testing for peripheral arterial disease. The ABI is an indispensable part of any foot examination. It is a simple bedside test also known as the Winsor index. It is the ratio of systolic blood pressure measured in the ankle to systolic blood pressure measured in the arm. Three readings of each are to be taken of which the highest value is to be selected. A ratio of 0.9-1.4 is considered to be normal. ABI between 0.6 and 0.9 denotes mild peripheral artery disease (PAD), 0.4-0.6 denotes moderate PAD and < 0.4 denotes severe PAD or critical limb ischemia (CLI). A value of more than or equal to 1.4 may also have the risk of having PAD which may be masked due to the presence of arterial wall calcification or atherosclerosis [30]. In these conditions, Toe Brachial Index can be calculated. It is the ratio of systolic blood pressure in the toe to systolic blood pressure in the arm. A ratio of > 0.6 is considered normal and toe blood pressure less than 30 mmHg is considered critical ischaemia. Another method to evaluate the perfusion of the lower limb is the transcutaneous partial pressure of oxygen (TcPO2). A value of > 60 mmHg denotes adequate perfusion of the lower limbs. A TcPO2 value of less than 30 mmHg signifies poor wound healing and the need for revascularisation procedures.

Diabetic foot ulcer

Patients with diabetic foot ulcers are classified based on the degree of ulcer depth, association with infection or ischaemia. Various grading systems for grading diabetic foot ulcers are present which include Meggitt-Wagner grading of diabetic foot ulcer, University of Texas grading, PEDIS (Perfusion, Extent, Depth, Infection, Sensation) system and SINBAD (Site, Ischaemia, Neuropathy, Bacterial infection, Area, Depth) system. They predict the risk of lower extremity amputation, morbidity and mortality. The most common clinically used classification systems like Meggitt-Wagner, University of Texas, PEDIS and SINBAD classification of foot ulcers are described below from Table 2 to Table 5 [31-34].

**Table 2:** Meggitt Wagner classification

Grade	Description
Grade 0	Skin intact but bony deformities lead to "foot at risk"
Grade 1	Superficial ulcer
Grade 2	Deep, full-thickness ulcer involving tendons but not bone
Grade 3	Involving the bone with abscess formation or osteomyelitis
Grade 4	Partial Gangrene of forefoot
Grade 5	Extensive Gangrene

**Table 3:** University of Texas classification

Grade	Description	Stage	Description
0	Pre-ulcerative epithelialized lesion	A	No infection or Ischemia
1	Superficial ulcer not involving tendon or bone	B	Infection present
2	Ulcer penetrating to tendon/capsule	C	Ischemia present
3	Ulcer penetrating to bone/joint	D	Infection and Ischemia present

**Table 4:** PEDIS (Perfusion, Extent, Depth, Infection, Sensation) classification PAD: peripheral artery disease; CLI: critical limb ischemia

Grade	Perfusion	Extent	Depth	Infection	Sensation
1	No PAD	Skin intact	Skin intact	None	Intact
2	PAD, no CLI	< 1 cm^2^	Superficial	Surface	Lost
3	CLI	1-3 cm^2^	Tendon	Abscess, septic arthritis	
4		> 3 cm^2^	Bone	SIRS	

**Table 5:** SINBAD classification SINBAD: Site, Ischaemia, Neuropathy, Bacterial infection, Area, Depth

Category	Description	Score
Site	Forefoot midfoot and hindfoot	0 1
Ischaemia	Pedal blood flow intact: at least one pulse palpable. Clinical evidence of reduced pedal blood flow	0 1
Neuropathy	Protective sensation intact: Protective sensation lost	0 1
Bacterial infection	None present	0 1
Area	Ulcer < 1 cm^2^ Ulcer > 1 cm^2^	0 1
Depth	Ulcer confined to the skin and subcutaneous tissue Ulcer reaching muscle, tendon or deeper	0 1
Total possible score		6

The ischaemic ulcer is seen predominantly in smokers and patients with dyslipidaemia and is characterised by claudication pain and rest pain in severe cases [35]. The affected limb has shiny skin, cool, pale and reduced hair growth and weak pulse with delayed capillary filling time [36]. The most common sites for ischemic ulcers are water-shed areas like the tips of toes, interdigital spaces and lateral margins of the foot. Ischemic ulcers are characterized by pale and punched-out edges, irregularly shaped, necrotic tissue and slough in the base of the ulcer with or without exudate. Gangrene may be seen associated with ulcers in severe peripheral arterial disease [37].

Venous ulcers are usually located above the medial malleolus. The ulcer is shallow, with heavy exudate and granulation tissue. Hyperpigmentation of the surrounding skin, normal pulses and normal capillary refilling time and oedema of the affected limb are seen.

Neuropathic ulcers are seen in patients with peripheral neuropathy with LOPS. The most common sites are pressure points like metatarsal heads, bony prominences and tips of the toes. The ulcer is deep, dry, painless and usually surrounded by a callus. The skin of the limb is dry and cracked. Foot muscles are atrophic with deformities like claw toes. Pulses are normal or hyperdynamic due to the loss of the sympathetic vasopressor effect.

To evaluate the involvement of bone in ulcers a common clinical test used is the Probe-To-Bone test. Here a sterile blunt metal probe is inserted into the ulcer and if a hard gritty part is felt then the test is considered to be positive which indicates the presence of bony involvement. The pooled sensitivity of this test was 0.87 (95% CI 0.75-0.93) and specificity was 0.83 (95% CI, 0.65-0.93) in a systematic review and meta-analysis. Thus, it can correctly diagnose the presence of osteomyelitis in high-risk [38]. Other tests for osteomyelitis workup include an X-ray foot, MRI foot and WBC scans. Haemogram, erythrocyte sedimentation rate and pus culture sensitivity can guide the management of infected ulcers.

X-ray foot in osteomyelitis reveals a break in the continuity of the cortex, the presence of periosteal reaction and subcutaneous tissue swelling. The presence of sequestrum which is dead necrosed bone coming out of the cortex and involucrum which is the new bone formation outside the previous periosteum are pathognomonic findings in osteomyelitis. It can be distinguished from active Charcot foot by the presence of sinus tracts and skin ulcerations. Active Charcot in the early stages may have a normal X-ray finding. Later stages in Charcot foot are characterised by the presence of debris, destructive bone lesions, bony dislocations, presence of subchondral fractures and demineralisation.

Footwear examination

Ill-fitting footwear can lead to frictional injury, blisters, calluses and ulcers leading to major issues. Properly fitting footwear can reduce foot problems and amputation by up to 85% [39]. Footwear should be examined at regular intervals for the presence of foreign body also. Patients should always be instructed to purchase their footwear in the evening, as the size of the feet is maximum due to the presence of swelling over the feet. The ideal shoe would be a snugly fitting shoe, not too tight and not too loose, with the presence of a wide toe box and enough height to accommodate the toes with or without any toe deformity. The insole of the shoe should be soft and it should be inspected for the presence of any pressure points or dents. In case of the appearance of pressure points on the insole, patients should be strictly instructed to change footwear. Patients with deformities should have custom-made shoes.

Risk evaluation

Risk stratification of the diabetic foot allows proper management and guides follow-up of the patient. The foot may be normal, with intact nerve function, normal blood flow and no deformity (risk zero), otherwise can be categorized as Risk 1, 2 or 3 depending upon the neuropathy status, blood flow and presence of or history of foot ulcers or deformity. Based on risk assessment, further management of the patient is suggested like footwear modification or even surgical procedures to prevent further complications. The duration of follow-up also depends on the risk category such as yearly, six monthly or three-monthly visits. According to the International Working Group on the Diabetic Foot (IWGDF), guidelines patients without any deformity or LOPS or PAD are classified as risk category 0 and are educated regarding foot care practices and appropriate footwear. They are suggested to follow up annually for foot evaluation. Patients with either LOPS or deformity are classified as risk category 1 and are advised prescriptive or accommodative footwear along with prophylactic surgery if deformity cannot be addressed safely by corrective footwear. These patients are followed up 3-6 monthly for foot evaluation. Patients with the presence of either LOPS or PAD are classified as risk category 2 and are advised prescriptive or accommodative footwear along with vascular surgery consultation and they are followed by 2-3 monthly. Patients with a history of foot ulcers or a history of amputation are classified as risk category 3 and are followed up monthly.

Foot care

Foot care education has a vital role in preventing foot problems. Instructions should be given regarding daily examination, proper foot hygiene, nail care, appropriate footwear along with socks even both indoors and outdoors and maintaining proper moisture of feet by using emollient. The importance of avoiding barefoot walking, any thermal exposure, chemical agents, self-treatments or self-surgeries should be clearly explained. Higher level multidisciplinary coordinated care involving medical management and foot care was found to be associated with lower amputation rates [40].

Glycemic control

Optimum glycemic control is paramount in the management of a diabetic patient. Uncontrolled hyperglycemia exacerbates neuropathy and vasculopathy which can lead to infection, deformity, ulceration or amputation. ADA recommended glycemic targets of HbA1C less than 7% in patients without risk of hypoglycemia or a less stringent target of less than 8% in selected patients should be achieved. Management of each patient is to be individualised and the decision to initiate insulin or oral antidiabetic drugs will be based on associated complications like nephropathy or cardiovascular disease. Good glycemic control is always necessary to prevent foot-related complications.

## Conclusions

Diabetic foot is a commonly neglected diabetic complication with far-reaching disabling consequences, terminating in amputation in a large number of cases. The risk of both major and minor amputation is increased in both types of diabetes, major amputation being much more common in type 2 diabetes mellitus. Type 2 diabetes is in fact the leading cause of foot amputation worldwide, almost six times more common than non-diabetic cause-related amputations. Management of diabetic foot includes preventive assessment and aggressive treatment. Individuals with diabetes should regularly visit foot care specialists. Daily self-examination by the patient, visual feet inspection during every clinic visit and annual comprehensive feet examination by the treating physician is recommended. Detailed annual feet exam including simple monofilament test, vibration and light touch assessment for neuropathy and palpation of peripheral pulses can help prevent amputation. Regular and comprehensive foot check-ups by the physician, good glycemic control, foot care and appropriate footwear all together will help in preventing diabetic foot problems which may ultimately help avoid amputation. Physicians, patients and caregivers all play an equal and important role in maintaining healthy feet. By training medical staff including nurses, medical students, postgraduate students and practising physicians about the basics of diabetic foot examination, both prevention and early detection can save patients from amputation. This review has provided a simplified ready reckoner for the specialists and primary care providers, commonly involved in managing diabetes-related foot complications. Already many organizations have taken initiatives in this direction and we hope to take it further by means of this article. So, KEEP TESTING - SAVE THE FEET - KEEP WALKING.
